# Brucine suppresses colon cancer cells growth *via* mediating KDR signalling pathway

**DOI:** 10.1111/jcmm.12108

**Published:** 2013-08-02

**Authors:** Wenjuan Luo, Xiaoli Wang, Lei Zheng, Yingzhuan Zhan, Dongdong Zhang, Jie Zhang, Yanmin Zhang

**Affiliations:** a School of Medicine, Xi'an Jiaotong UniversityXi'an, Shaanxi Province, China

**Keywords:** Brucine, Angiogenesis, LoVo cells, KDR, Phosphorylation

## Abstract

Angiogenesis plays an important role in colon cancer development. This study aimed to demonstrate the effect of brucine on tumour angiogenesis and its mechanism of action. The anti-angiogenic effect was evaluated on the chicken chorioallantoic membrane (CAM) model and tube formation. The mechanism was demonstrated through detecting mRNA and protein expressions of VEGFR2 (KDR), PKCα, PLCγ and Raf1 by reverse transcription-polymerase chain reaction (RT-PCR) and Western blot (WB), as well as expressions of VEGF and PKCβ and mTOR by ELISA and WB. The results showed that brucine significantly reduced angiogenesis of CAM and tube formation, inhibited the VEGF secretion and mTOR expression in LoVo cell and down-regulated the mRNA and phosphorylation protein expressions of KDR, PKCα, PLCγ and Raf1. In addition, the effects of brucine on KDR kinase activity, viability of LoVo cell and gene knockdown cell were detected with the Lance™ assay, WST-1 assay and instantaneous siRNA. Compared to that of normal LoVo cells, the inhibition on proliferation of knockdown cells by brucine decreased significantly. These results suggest that brucine could inhibit angiogenesis and be a useful therapeutic candidate for colon cancer intervention.

## Introduction

Angiogenesis refers to the process of capillary formation from pre-existing blood vessels and plays an important role in the growth and spread of cancer [1, 2]. Tumour cells promote vessel formation through the expression of angiogenic molecules or their induction in the microenvironment [3]. As a target for cancer chemotherapy, blocking angiogenesis could be a strategy to arrest tumour growth [4]. VEGF is the best-characterized angiogenic cytokine and the most potent angiogenesis inducer [5]. VEGF binding to its receptor (VEGFR) leads to cell proliferation and new vascular formation by the tyrosine kinase (TK) pathway. The VEGF/VEGFR pathway therefore becomes an attractive target for anticancer drug design [6]. KDR (VEGFR2) is the predominant receptor in angiogenic signalling. Its activation regulates endothelial cell migration, proliferation, differentiation, survival as well as vessel permeability and dilation. There are various molecular players and signalling cascades involved in the VEGF/VEGFR pathway, such as the phosphatidylinositol 3-kinase (PI3K)/AKT, Ras/Raf/mitogen-activated protein kinase (MAPK) and phospholipase-Cγ/protein kinase C (PLCγ/PKC) pathway. These signalling pathways regulate important cellular functions including cellular proliferation, migration, angiogenesis and apoptosis [7–10].

**Fig. 1:**
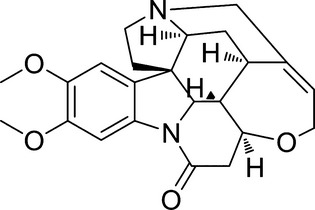
Chemical structure of brucine.

Brucine (Fig. 1), an indole alkaloid, is isolated from seeds of strychnos nux-vomica L. (Loganiaceae), a traditional medicinal herb, native to East India, Burma, Thailand, China and Northern Australia. In previous reports, it has been used for the treatment of analgesia, diabetes, anaemia, gonorrhoea, anti-inflammation and anti-cancer [11–13]. For instance, Saraswati *et al*. and Li *et al*. reported that brucine could inhibit angiogenesis by HUVECs, and microvessel density and bone metastasis by breast cancer [14, 15]. Brucine is always in its active conformation that binds to and activates KDR [16]. We have previously reported that ERK1/2 and AKT could be down-regulated by brucine. So the KDR and main pathway molecules, PKCα, PLCγ, Raf1, PKCβ and mTOR, were selected for the mechanism research of suppressing colon cancer cell growth by brucine.

By the docking study, we found that there was a good interaction between brucine and KDR. This study aimed to extend the previous study of brucine and to evaluate its inhibition on colon cancer cell growth through mediating KDR and its signalling pathway of phosphorylation. All the results suggest that brucine could inhibit colon cancer cell growth by anti-angiogenesis and be a useful therapeutic candidate for colon cancer intervention.

## Materials and methods

### Materials

Brucine was from the Shaanxi institute for food and drug control, the purity of which was over 99.5%. Trypsin and fibrinogen were from Sigma-Aldrich (St. Louis, MO, USA). Human VEGF was from Peprotech Asia (Rehovot, Israel). WST-1, protease inhibitor cocktail and phosphatase inhibitor cocktail were from Roche (Roche Tech., Mannheim, Germany). Anti-phospho-KDR (Tyr^1175^) was from Cell Signaling (Cell Signaling Tech., Danvers, MA, USA), anti-phospho-PKCα (Tyr^658^) and anti-phospho-PLCγ-1 (Tyr^771^) were from Upstate (Upstate, Temecula, CA, USA), anti-phospho-Raf1 (Tyr^340/Tyr341^) was from Millipore (Millipore, Billerica, MA, USA). Rabbit anti-GAPDH was from Santa Cruz Biotech (Dallas, TX, USA). Rabbit anti-PKCβ and anti-mTOR were from proteintech group (Chicago, IL, USA). Rabbit antimouse IgG, goat anti-rabbit IgG, BCA protein assay reagent kit and enhanced chemiluminescent (ECL) plus reagent kit were obtained from Thermo (Thermo, Rockford, IL, USA). Total RNA extraction kit was from Fastagen (Fastagen, Shanghai, China). Revert AID™. first strand cDNA synthesis kit and RNAi were from Fermentas (Hanover, Lithuania). Other reagents used were analytical grades.

### Cell culture

LoVo cells from Shanghai Institute of Cell Biology in the Chinese Academy of Sciences were maintained in RPMI1640 with 10% FBS. HUVEC from ATCC was cultured in F-12K media supplemented with 0.1 mg/ml heparin, 0.05 mg/ml endothelial cell growth supplement (ECGS) and 10% FCS. Antibiotics (100 IU/ml penicillin and 100 μg/ml streptomycin), at 37°C in a 5% CO_2_ atmosphere.

### CAM assay

Chorioallantoic membrane was prepared as described [17]. Briefly, a circular window, ∼1.5–2 cm in diameter, was opened aseptically on the egg shell. A quantity of 10 μl brucine was added to the CAM surface in every egg. Doses of 5, 10 and 20 μg/egg were used here. At least 10 eggs were used for each sample dose. The embryos were further incubated for 72 hrs after administration. The anti-angiogenic response was assessed by counting. Five randomly chosen fields were evaluated for each specimen. The total number of large vessels, small vessels and capillaries in the fields were counted. The inhibitory effect on blood vessels could be observed by comparing the vascular change between the brucine group and the negative control group.

### Tube formation assay

A 48-well plate coated with 0.2 ml matrigel per well was allowed to solidify at 37°C for 0.5–1 hr. Each well was seeded with 5 × 10^4^ HUVEC cells and cultured in F-12K containing 50 ng/ml VEGF at various concentrations of brucine or vehicle alone. The enclosed networks of tubes were photographed from five randomly chosen fields under a microscope.

### Docking study

In an effort to elucidate the binding modes of brucine with KDR, it was constructed with Sybyl/Sketch module and optimization was performed with Powell's method with the Tripos force field with convergence criterion set at 0.05 kcal/(Å mol), and assigned with Gasteiger-HŰckel method [18]. The docking study was performed with Sybyl/Surflex module, and the residues in a radius of 6.5 Å around the (PDB ID: 1IVO) were selected as the active site. Other docking parameters implied in the program were kept default.

### Lance™ assay for KDR kinase activity

KDR kinase was determined by Lance™ assay. 2 μl kinase (Carna Biosciences, Kobe, Japan) and 2 μl substrate was added to the 384-well plate, and brucine at various concentrations was then added to the assay plate. 2 μl ATP was added and the reaction was allowed to proceed at 37°C for 30 min. The TK-antibody labelled with Eu^3+^-cryptate and streptavidin-XL665 was then added with EDTA to detect the phosphorylated product at room temperature for 1 hr. Then the fluorescence measurements of the resulting solution at 665 and 615 nm were performed with the plate reader of Perkin-Elmer victor 5. The kinase activity was expressed by the ratio of A665 × 10^4^/A615 [7].

### Reverse transcription quantitative real-time PCR

Isolation of total RNA of LoVo cells treated with or without brucine was performed with a total RNA extraction kit. Reverse transcription of total RNA in 20 μl reaction solution was performed with the Revert AID™ first strand cDNA synthesis kit. Its integrity and subsequent RT-PCR performed for KDR, PKCα, PLCγ, Raf1 and GAPDH are described previously [19, 20]. The sequence details of individual pairs of primers of KDR, PKCα, PLCγ, Raf1 and GAPDH are in Table 1. The PCR reactions were performed with the Thermal Cycler Dice Real Time System (Takara, Kyoto, Japan) on 96-well reaction plates. The relative amount of mRNA for each gene was normalized and represented as the ratio of the mRNA value of a target gene to that of the β-actin gene.

**Table 1:** Sequence details of individual pairs of primers

Gene	Sense	Antisense
KDR	5′-GAGTGAGGAAGGAGGACGAAGG-3′	5′-CCGTAGGATGATGACAAGAAGTAGC-3′
PKCα	5′-ATGGCGTCCTGTTGTATGAAATGC-3′	5′-GGTGTTTGGTCATCAGTCCTTTGC-3′
PLCγ-1	5′-GAGGAGGCACTGGAGAAGATTGG-3′	5′-GCACACTTGAAAGTTGGCATAGGG-3′
Raf1	5′-TCTACACCTCACGCCTTCACC-3′	5′-CATCCTCAATCATCCTGCTGTCC-3′
GAPDH	5′-CACCCACTCCTCCACCTTTG-3′	5′-CCACCACCCTGTTGCTGTAG-3′

### Western blot analysis

The LoVo cells treated with or without brucine for 48 hrs were prepared by extracting proteins with RIPA lysis buffer containing a protease inhibitor cocktail and a phosphatase inhibitor cocktail on ice. Cell lysates were analysed for Western blot analysis with primary antibodies [KDR(Tyr^1175^), PKCα (Tyr^658^), PLCγ-1(Tyr^771^) and Raf1(Tyr^340/341^)], followed by enhanced chemiluminescence [21, 22]. At the same time, PKCβ and mTOR expressions were also determined. Analysis of the protein expression was performed with Quantity one®, 1-D analysis software (Version 4.4, BioRad, Hercules, CA, USA).

### VEGF secretion *in vitro*

LoVo cells (1 × 10^4^ cells per well) were cultured in 24-well culture plates for 24 hrs. Then, the cells were incubated for another 24 hrs after the medium was changed to a serum-free medium. The same volume with different brucine concentrations was added to the wells respectively. The 48-hr cultured medium was collected. VEGF protein concentrations were quantitatively measured by a commercially available VEGF-ELISA kit at 450 nm [23].

### siRNA transfections

For *in vitro* knockdown experiments, a smart pool of double-stranded siRNA against KDR, PKCα, PLCγ and Raf1 as well as non-specific siRNA was obtained from Shanghai GenePharma Co. Ltd. siRNA was delivered at a final concentration of 50 nM and transfection was performed with Lipofectamine 2000 reagent (Invitrogen, Carlsbad, CA, USA) according to the manufacturer's instructions [24, 25]. The sense and antisense sequences are in Table 2. We incubated the cells for 24 hrs to allow knockdown of KDR, PKCα, PLCγ and Raf1. These cells were used for proliferation assays.

**Table 2:** Designed and synthesized a double-stranded siRNA oligonucleotide

Gene	Sense	Antisense
KDR	5′-GGCAUGUACUGACGAUUAUTT-3′	5′-AUAAUCGUCAGUACAUGCCTT-3′
	5′-CCGGGAUAUUUAUAAAGAUTT-3′	5′-AUCUUUAUAAAUAUCCCGGTT-3′
	5′-GUCCCUCAGUGAUGUAGAATT-3′	5′-UUCUACAUCACUGAGGGACTT-3′
PKCα	5′-GUCCCAUGAAUUUGUUACUTT-3′	5′-AGUAACAAAUUCAUGGCACTT-3′
	5′-GAGUCCUUUACAUUCAAAUTT-3′	5′-AUUUGAAUGUAAAGGACUCTT-3′
	5′-GCGUCCUGUUGUAUGAAAUTT-3′	5′-AUUUCAUACAACAGGACGCTT-3′
PLCγ-1	5′-CCCUGCUGAUCAAGAUUGATT-3′	5′-UCAAUCUUGAUCAGCAGGGTT-3′
	5′-GGGACUUUGAUCGCUAUCATT-3′	5′-UGAUAGCGAUCAAAGUCCCTT-3′
	5′-GUGCCUUUGAAGAACAACUTT-3′	5′-AGUUGUUCUUCAAAGGCACTT-3′
Raf1	5′- GCAGGUUGAACAACCUACUTT -3′	5′- AGUAGGUUGUUCAACCUGCTT -3′
	5′- GCACCAAAGUACCUACUAUTT -3′	5′- AUAGUAGGUACUUUGGUGCTT -3′
	5′- CCCACACUGAGGAUAUCAATT -3′	5′- UUGAUAUCCUCAGUGUGGGTT -3′
Negative control	5′-UUCUCCGAACGUGUCACGUTT-3′	5′-AGGUGACACGUUCGGAGAATT-3′

### Proliferation assay

LoVo cells and cells of siRNA transfections (1 × 10^4^) were cultured in 96-well microtitre plates and fresh medium with or without brucine was added for 48 hrs. Cell proliferation reagent WST-1 was added and incubated at 37°C and 5% CO_2_ for 1 hr. Absorbance was then measured at 440 nm with a microplate reader (Bio-Rad instruments, USA).

### Data analysis

Data are given as mean ± SD in quantitative experiments. Statistical analyses of differences between the groups were performed with anova by Student's *t*-test. A *P*-value less than 0.05 was considered statistically significant.

## Results

### Brucine inhibited the CAM angiogenesis and tube formation

**Fig. 2:**
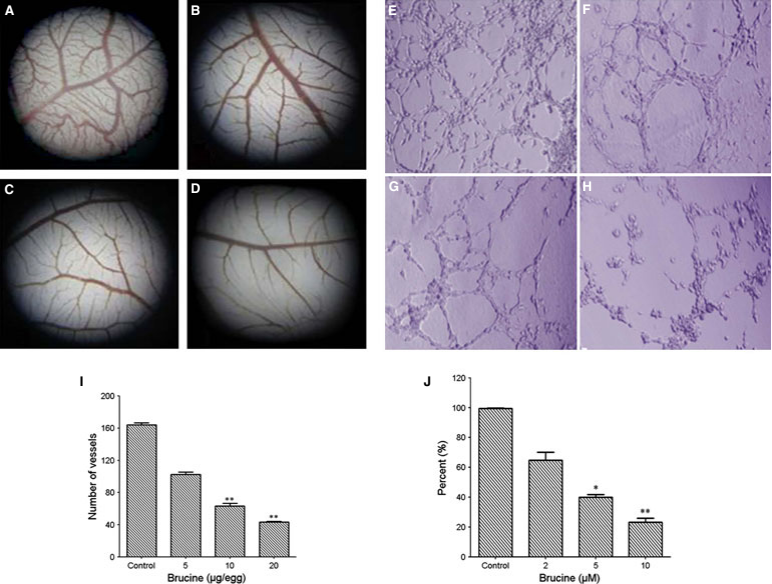
Effect of brucine on CAM angiogenesis and tube formation. (**A**–**D**) Effect of brucine on the CAM (magnification 40 ×). In the negative group, the blood vessels grew normally. Treated with various concentrations (5, 10, 20 μg/egg) of brucine, the inhibitory effect on CAM blood vessels seemed increasingly obvious. (**A**) Negative control. (**B**) Brucine, 5 μg/egg. (**C**) Brucine, 10 μg/egg. (**D**) Brucine, 20 μg/egg. (**E**–**H**) Effect of brucine on the tube formation (magnification 40 ×). In the untreated control group, cells formed the tube in the matrigel. In the brucine group, formed tube was inhibited obviously in dose-dependent manner. (**E**) Negative control. (**F**) Brucine, 2 μM. (**G**) Brucine, 5 μM. (**H**) Brucine, 10 μM. (**I**) Quantitation data of (**A**–**D**). (**J**) Quantitation data of (**E**–**H**). Compared with the negative control group, brucine showed the good inhibitory effect on the CAM angiogenesis and tube formation in a good dose-dependent manner. Data are expressed as means ± SD (*n* = 5). *P < 0.05, **P < 0.01 *versus* the untreated control group.

Chorioallantoic membrane angiogenesis model and tube formation assay were used for testing the effect of brucine on angiogenesis, compared with the control, brucine inhibited the CAM angiogenesis within the concentration range of 5–20 μg/egg. In the negative group, the blood vessels grew normally, new capillary vessels were generated, the density and area of the CAM blood vessels tended to increase. However, in the brucine-treated group, no new capillary vessels were generated and angiogenesis was inhibited in a good dose-dependent manner (Fig. 2A–D and I). In addition, HUVEC cells were induced by VEGF and treated with vehicle or brucine. HUVEC cells were incubated on matrigel with VEGF, forming an extensive and enclosed network of tubes. Brucine significantly decreased the number of the tube structure (Fig. 2E–H and J) at the concentrations of 2–10 μM respectively.

### Interaction by docking study

**Fig. 3:**
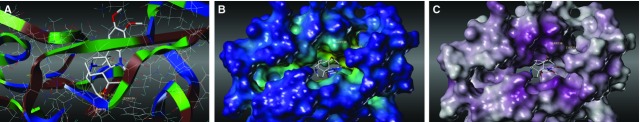
Docking simulation of brucine with KDR (PDB ID 1IVO) was carried out with Surflex. (**A**) Two H-bond interactions between oxygen atom of brucine and amino acid residues of the receptor. Hydrogen bonds between brucine and the residues are shown with yellow dotted lines; (**B**) Molcad surface cavity depth; (**C**) Molcad surface H-acceptor/donor density.

Docking of brucine in the active site of KDR showed two H-bond interactions between the oxygen atom of brucine and amino acid residues of the receptor (Fig. 3A). According to the docking simulation, the oxygen formed two hydrogen bonds to ASP238 and THR239 with distances of 1.96 and 2.71 Å respectively. We also could predict that brucine displayed a good fit with the KDR receptor domain which was not occupied by small molecular RTK inhibitors (Fig. 3B and C). The simulated binding mode was in concordance with experimental results. This binding hypothesis may provide valuable information for the structure-based design for brucine derivatives acting as potent anticancer agents. As seen from Figure 3B, brucine could occupy a critical binding pocket of KDR which was possibly essential for the interaction with EGF. Figure 3C indicated the hydrogen bond density on the surface of the receptors. All the above findings showed that brucine had good action on KDR.

### Brucine suppresses the VEGF secretion and PKCβ and mTOR expressions

**Fig. 4:**
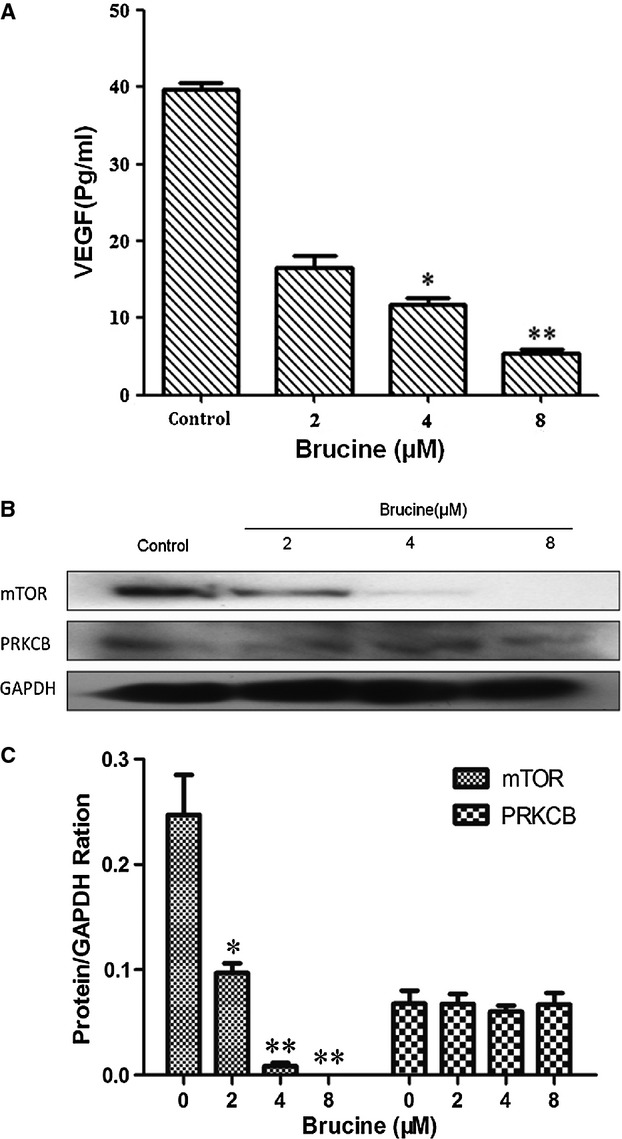
Effect of brucine on VEGF, PKCβ and mTOR expressions. (**A**) VEGF expressions were inhibited in a dose-dependent manner compared with the control group. (**B**) Effect of brucine on PKCβ and mTOR expressions. (**C**) Quantitation data of (**B**). Results were quantified by densitometry analysis of the bands form and then normalization to GAPDH protein. Data represent the means ± SD (*n* = 3) with **P < 0.01 *versus* the untreated control.

ELISA for VEGF showed that brucine could inhibit VEGF production in a dose-dependent manner compared with the control group in LoVo cells (*P* < 0.05). The VEGF expressions clearly decreased at different concentrations (Fig. 4A). There were significant differences between the brucine group and the control group. In addition, brucine inhibited the mTOR expression and did not show obvious inhibition on PKCβ (Fig. 4B and C).

### Effect of brucine on KDR kinase

The Lance™ assay was used to assess the effect of brucine on KDR kinase activity. The optimized used concentrations of reaction system were as follows: KDR kinase 0.0038 ng/μl, ATP 1.33 μM and substrate 121.40 nM respectively. The IC50 of brucine on KDR kinase activity was over 5000 nM, suggesting that brucine did not alter KDR kinase activity effectively.

### Effect of brucine on mRNA of KDR signalling pathway of phosphorylation

**Fig. 5:**
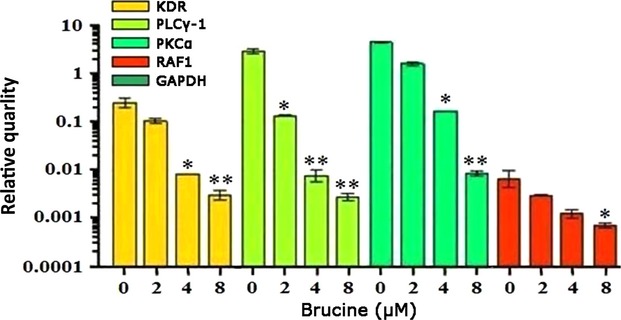
Effect of brucine on mRNA expressions of KDR, PKCα, PLCγ and Raf1 in LoVo cells. Relative ratio is shown, where KDR, PKCα, PLCγ and Raf1 signals were normalized to GAPDH signal. Data represent the means ± SD (*n* = 3) with *P < 0.05, **P < 0.01 *versus* the untreated control.

Semi-quantitative PCR was carried out to understand whether brucine could influence synthesis of KDR, PKCα, PLCγ and Raf1 transcript. As shown in Figure 5, the mRNA levels of KDR, PKCα, PLCγ and Raf1 in the brucine-treated group were significantly down-regulated in a dose-dependent manner compared with the negative control (*P* < 0.05). It indicated that brucine could regulate the mRNA levels of KDR, PKCα, PLCγ and Raf1.

### Effect of brucine on KDR signalling pathway of phosphorylation proteins

**Fig. 6:**
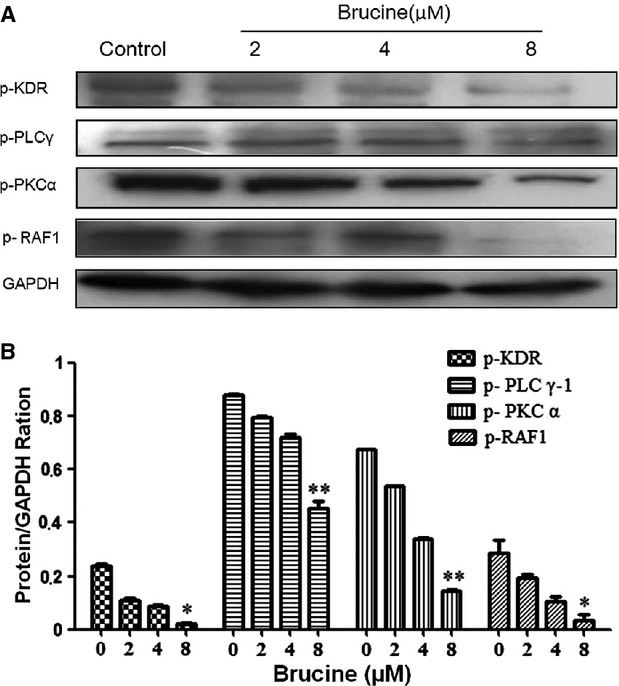
Effect of brucine on protein expressions of KDR, PKCα, PLCγ-1 and Raf1 in LoVo cells. Cells were treated with VEGF (50 ng/ml) for 30 min. before extracting proteins with RIPA lysis buffer. Results were quantified by densitometry analysis of the bands form and then normalization to GAPDH protein. (**A**) Effect of brucine on protein expressions. (**B**) Quantitation data of (**A**). Quantitation data showed brucine decreased the phosphorylation levels of KDR, PKCα, PLCγ-1 and Raf1 in a dose-dependent manner compared to the untreated control. Data represent the means ± SD (*n* = 3) with *P < 0.05, **P < 0.01 *versus* the untreated control.

To further identify the effect of brucine on KDR and to assess whether brucine altered the signalling pathways that might have contributed to growth inhibition, this study tested the phosphorylation status of KDR(Tyr^1175^), PKCα (Tyr^658^), PLCγ-1(Tyr^771^) and Raf1 (Tyr^340^/Tyr^341^) in LoVo cell by Western blot analysis. Brucine significantly reduced the phosphorylation of KDR, PKCα, PLCγ-1 and Raf1 (Fig. 6A). Figure 6B shows the quantitation of protein expressions. These results suggest that the activated KDR and its downstream signalling pathways proteins could be down-regulated by brucine in a dose-dependent manner.

### Proliferation inhibition of brucine on LoVo cells and cells of gene knockdown

**Fig. 7:**
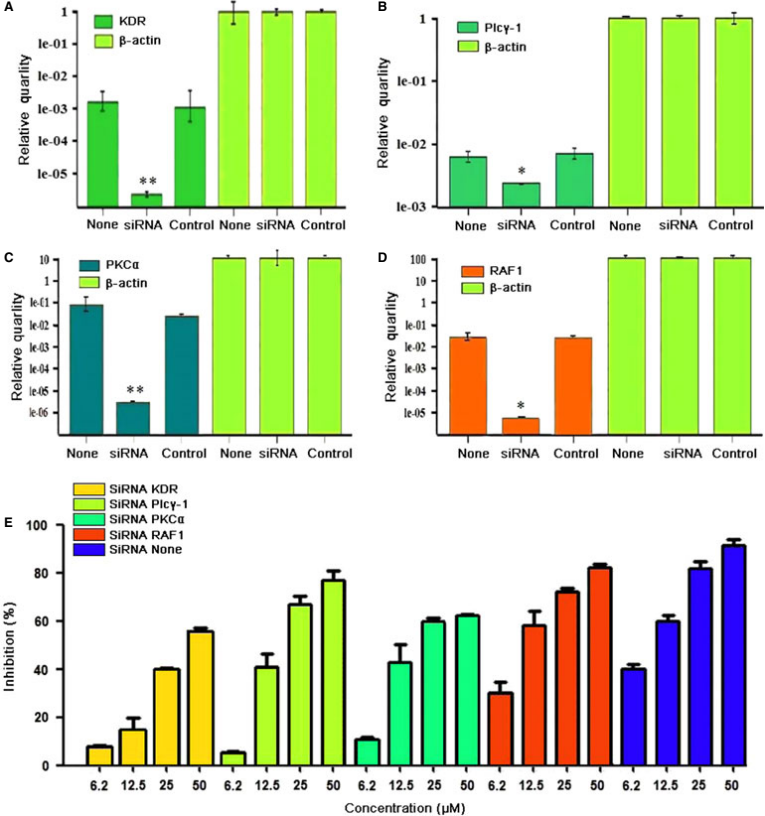
Effect of brucine on normal cells and cells transfected with siRNAs of KDR, PKCα, PLCγ and Raf1. (**A**–**D**) Knockdown quantification of RT-PCR on KDR, PKCα, PLCγ and Raf1; (**E**) The effect of brucine on cells proliferation of normal cells and knockdown cells. Data are expressed as means ± SD (*n* = 3).

To further assess the roles of KDR, PKCα, PLCγ-1 and Raf1 phosphorylation in cancer cell growth, siRNA-targeting these proteins were transfected into cells (Fig. 7A–D) followed by brucine treatment for 48 hrs. Compared with the untransfected cells, inhibition of brucine on LoVo cells transfected with siRNA targeting KDR, PKCα, PLCγ-1 and Raf1 decreased obviously respectively (Fig. 7E). It indicated that the inhibition on untransfected cells was better than the cells of gene knockdown.

## Discussion

Previous studies showed that brucine inhibited VEGF expression and decreased microvessel density in a nude mouse model of bone metastasis because of breast cancer [15], and brucine could inhibit cell proliferation, chemotactic motility and down-regulate levels of VEGF, NO, IL-6, IL-8, TNF-α and IFN-γ in HUVECs [14]. However, there have been no reports about its inhibition on colon cancer growth. The results in this study indicated that the decreased LoVo cell growth by brucine was accompanied by diminished angiogenesis. Angiogenesis has become an attractive target for drug therapy because of its key role in tumour growth, and inhibition of angiogenesis provides a good chance of preventing cancer from becoming malignant [7, 26]. A large body of evidence indicates that the VEGF/VEGFR system is involved in angiogenesis. Angiogenesis inhibitors targeting VEGF or VEGFR are among the largest group of anticancer agents that are currently being explored in clinical studies [27].

In this study, the CAM assay and tube formation model showed that brucine could inhibit CAM angiogenesis and tube formation of HUVEC, and inhibit the VEGF protein secretion. Then, we examined the mechanisms associated with anti-angiogenic activity of brucine. Brucine acted on KDR and inhibited the phosphorylation of KDR. However, the IC50 on KDR kinase was over 5000 nM in LanceTM assay for KDR kinase activity. A similar result in a recent report has shown that the IC50 of VEGFR2 kinase by brucine was 21.34 μM (>5000 nM) [14]. Generally, the above data show that VEGFR kinase activity is not affected by brucine, indicating that the inhibition of KDR phosphorylation is not because of inhibition on KDR kinase.

The various downstream signalling molecular players, such as PI3K/AKT, MAPK and PLCγ/PKC, have the specific functions including cellular proliferation, migration, angiogenesis and apoptosis [9, 10]. PLCγ-1, a very important member of phospholipase-C (PLC) families, is up-regulated in many cancer tissues and cancer cell lines and has been found to participate in many physical processes [28, 29]. There is an intimate relation between PLCγ-1 and PKCα. A decrease in the extent of tyrosine phosphorylation of PLCγ-1 has also been proved to be positively regulated by PKCα [30, 31]. Furthermore, in the MAPK/ERK pathway Raf1 becomes activated when it binds to Ras [32], several MAPK kinases have been suggested to be important for phosphorylation of Raf1 as well as positive feedback phosphorylation by MAPK. This observation represents a conformation in which Raf1 can phosphorylate the downstream target MEK, and this allows Raf1 to function as part of a kinase cascade [33, 34]. Mammalian target of rapamycin (mTOR) is a key kinase acting downstream of the activation of PI3K [35], and mTOR that acts as a master switch of cellular catabolism and anabolism determining tumour cell growth and proliferation [36]. Results from the RT-PCR and WB assay showed that brucine inhibited the mRNA expressions of PKCα, PLCγ and Raf1, and their phosphorylation. Furthermore, brucine inhibited the VEGF secretion and mTOR expression, but had no obvious inhibition on PKCβ. It indicated that brucine inhibited LoVo cell growth and angiogenesis by targeting signalling molecules PKCα, PLCγ-1 and Raf1, which was confirmed by the subsequent siRNA assay. Knockdown of KDR, PKCα, PLCγ and Raf1 by siRNA significantly attenuated tumour inhibitory effects of brucine.

In conclusion, this study extends the study of brucine and suggests that brucine has an anti-angiogenic activity through down-regulating the phosphorylation signalling of KDR, PKCα, PLCγ-1 and Raf1. All the results from this study make it possible that brucine acts as a potential angiogenesis inhibitor and a useful therapeutic candidate for colon cancer intervention.
